# On the effective depth of viral sequence data

**DOI:** 10.1093/ve/vex030

**Published:** 2017-11-14

**Authors:** Christopher J R Illingworth, Sunando Roy, Mathew A Beale, Helena Tutill, Rachel Williams, Judith Breuer

**Affiliations:** 1 Department of Genetics, University of Cambridge, Cambridge, UK; 2 Department of Applied Maths and Theoretical Physics, Centre for Mathematical Sciences, University of Cambridge, Cambridge, UK; 3 Division of Infection and Immunity, University College London, London, UK; 4 Wellcome Trust Sanger Institute, Hinxton, Cambridge, UK

**Keywords:** population genetics, sequence data, evolutionary modelling

## Abstract

Genome sequence data are of great value in describing evolutionary processes in viral populations. However, in such studies, the extent to which data accurately describes the viral population is a matter of importance. Multiple factors may influence the accuracy of a dataset, including the quantity and nature of the sample collected, and the subsequent steps in viral processing. To investigate this phenomenon, we sequenced replica datasets spanning a range of viruses, and in which the point at which samples were split was different in each case, from a dataset in which independent samples were collected from a single patient to another in which all processing steps up to sequencing were applied to a single sample before splitting the sample and sequencing each replicate. We conclude that neither a high read depth nor a high template number in a sample guarantee the precision of a dataset. Measures of consistency calculated from within a single biological sample may also be insufficient; distortion of the composition of a population by the experimental procedure or genuine within-host diversity between samples may each affect the results. Where it is possible, data from replicate samples should be collected to validate the consistency of short-read sequence data.

## 1. Introduction

Genome sequencing provides a powerful tool with which to study pathogen evolution (Biek et al. 2015). The high evolutionary rates observed in many pathogens enable the observation of evolutionary changes over short periods of time. For example, serial sampling of within-host HIV populations shows a rapid accumulation of substitutions in the population over time (Shankarappa et al. 1999; Zanini et al. 2015). The rate of such change allows for clinically valuable information to be derived via a combination of genome sequencing and phylogenetic methods (Leitner et al. 1996). Whole-genome sequencing is anticipated to have a substantial impact on clinical virological practice (Houldcroft et al. 2017).

In some situations, substitutions in viral populations occur more slowly than can be resolved using consensus genome sequences. Influenza infections are typically short in duration, so that viral populations may retain a conserved consensus sequence across multiple transmission events (Murcia et al. 2012). In such cases, statistical approaches using the existence and frequency of variant alleles may be applied to infer the existence of transmission events and the presence of within-host selection from the data (Stack et al. 2012; Illingworth et al. 2014). With the advance of sequencing technologies such statistics are increasingly obtainable from viral populations, and are being increasingly utilised to generate insights into the dynamics of viral growth (Neher and Leitner 2010; Ganusov et al. 2011; Acevedo et al. 2014; Visher et al., 2016; Debbink et al. 2017; Sobel Leonard et al. 2017) and transmission (Wilker et al. 2013; Moncla et al. 2016; Poon et al. 2016).

Where a statistical model is fitted to genome sequence data, it is important either that the data used in the model fitting are accurate, or that inaccuracies in the data are properly accounted for. Noise in genome sequence data may arise from a number of sources (Beerenwinkel and Zagordi 2011; van Dijk et al. 2014; Laehnemann et al. 2016; Sandmann et al. 2017). For example, the extent of information available in a sample is limited by the quantity of biological material the sample contains prior to any amplification; this quantity can be estimated by limiting dilution (Rodrigo et al. 1997). The effect of resampling of a finite set of molecules can be modelled in a straightforward manner (Liu et al. 1996); a recent study of HIV evolution incorporated the effects of limited template number into an analysis of the fitness effects of viral mutations (Zanini et al. 2017). Amplification of biological material via polymerase chain reaction (PCR) can distort the content of a sample, proportionally increasing the fraction of some short reads compared to others (Kebschull and Zador 2015). The challenge of such distortions has been highlighted in identifying 16S sequence diversity in bacterial populations (Pinto et al. 2012); approaches which reduce sequencing noise have been developed for such cases (Quince et al. 2009; Schloss et al. 2011). PCR-based methods have been documented as giving rise to false variant calls (Varghese et al. 2010).

Variant calling involves the identification of single nucleotide polymorphisms (SNPs) in the population and is a key step in the evolutionary analysis of sequence data. A variety of approaches have been developed to identify true SNPs from genomic data, using routines that correct for sequencing errors (Li and Durbin 2009; Archer et al. 2010; Salmela and Schroder 2011; Macalalad et al. 2012; Iyer et al. 2013). In experimental studies, efforts are frequently made to determine a minimum threshold above which SNPs may be identified with confidence, for example, via the sequencing of plasmids of known sequence (Wang et al. 2007; Foll et al. 2014; Zanini et al. 2015). Errors in SNP calling have been identified as affecting population genetic statistics calculated from sequence data, such as the estimated diversity of a viral population (McCrone et al. 2016).

Having considered the presence or absence of SNPs, the frequency at which extant SNPs exist is also important for evolutionary analysis. Different approaches for validating the frequency of SNPs in a population have been presented. Considering HIV populations, a comparison of allele frequencies generated by short-read and Sanger sequencing methods validated the latter as an approach for studying early infection (Iyer et al. 2015). Where sections of a virus have been sequenced in independent PCR reactions, the frequencies of minority alleles in sections of overlap between fragments are calculated twice, allowing for the independent measurement of some allele frequencies (Zanini et al. 2016). In this study, a close link was found between the number of templates in a sample and the precision with which allele frequencies are identified. Primer IDs have been used as an alternative method to validate allele frequency calls for an HIV population (Jabara et al. 2011; Seifert et al. 2016). Measures such as these describe the extent to which independent measures of a sample produce consistent results.

**Figure 1.**
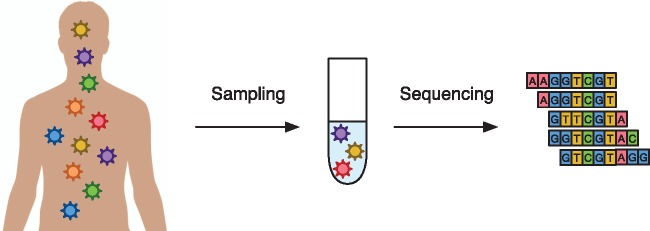
Pathways via which a short-read dataset may not accurately represent a within-host population. A viral sample collected from a patient may contain a population of viruses that do not fully represent the genetic diversity of the population within the host. Further, when sequenced, the output data may provide a distorted view of the material contained within the sample.

In the examples given above, independent measures collected from a single sample have been used to validate the extent to which noise during the sequencing process has induced variance in the data. However, we note that such approaches are incomplete in so far as they ignore sample bias, whereby the collected sample is unrepresentative of the within-host viral population; noise in a dataset may arise either from the unrepresentative sampling of material from a host, or from the subsequent processing of this material (Fig. 1). Many common viruses, including influenza (Hamada et al. 2012; Lakdawala et al. 2015; Sobel Leonard et al. 2017), HIV and related viruses (Ait-Khaled et al. 1995; Rose et al. 2016; Feder et al. 2017), and cytomegalovirus (CMV) (Renzette et al. 2013), have been identified as existing as separate subpopulations within a host, which may exhibit distinct viral diversity. In so far as mathematical models of viral evolution fit a model population to data from genome sequencing, failure to consider this structure may lead to overconfidence in the extent to which a model reproduces the true within-host viral population.

In order to investigate the relative importance of within- and between-sample variance in genome sequence data, we carried out replicate sequencing of material from single clinical samples from HIV, norovirus, and HCV infected patients. We further sequenced material from replicate samples of cerebrospinal fluid from two HSV1-infected patients. Differences between datasets were measured using a statistic comparing allele frequencies derived from each set, similar to the effective population size often calculated in population genetic studies. The effective population size describes the change in allele frequency expected through random drift in an idealised population of given size (Wright 1931; Felsenstein 1971; Charlesworth 2009); calculations comparing allele frequencies have been used to estimate population size and transmission bottlenecks in viral populations (Maldarelli et al. 2013; Poon et al. 2016). Measurements of the variance between allele frequencies have been used to calculate an ‘effective template number’ characterizing the internal accuracy of data in a viral genome sample (Zanini et al. 2015); here, we adopt a maximum likelihood approach for estimating noise in genome sequence data to calculate a similar ‘effective depth’ of sequencing (Illingworth 2015). Our statistic, while mathematically distinct from that of Zanini et al., shares the common property of fitting a variance to allele frequency data; our interest here is in the comparative measurement of this statistic across different sets of viral data.

We evaluated the extent of consistency between SNP calls in our sequenced data, and in data from an additional, previously published dataset, describing within-host diversity in influenza populations (Lakdawala et al. 2015). Across our data, we identifed a broad range of results, showing that within- and between-sample variance may both be significant factors in genomic datasets. Where independent sequencing runs were derived from same-sample replicates that had undergone identical processing before sequencing the consistency between runs was high. However, differences in the processing of a sample, or the processing of different samples from the same individual, did not always give such high consistency. A high absolute read depth of sequencing and a high number of templates in a sample do not guarantee that a viral population is accurately reproduced; a more nuanced approach to uncertainty in viral sequence data is required.

## 2. Results

We compare replicate genome sequence datasets in terms of the difference between the allele frequencies observed in each set. If a large and representative sample of viruses were collected from a population, and sequenced via an error-free process, the precision with which an allele frequency could be estimated depends upon the depth of reads via which the frequency is measured. For example, given an allele frequency of 20% measured via a binomial distribution, the standard deviation in the estimate is ±4% at 100× read coverage, but only ±0.4% at a read depth of 10^4^. Given the presence of error in sequencing, or unrepresentative sampling of a population, this variance would be increased. In simple terms, our ‘effective depth’ may be considered as the depth of an idealised sequencing process that would give an amount of variance equal to that observed in the data.

Mathematically, our statistic is calculated in two steps. First, in each case where two alleles are observed at a single locus, a beta-binomial distribution with parameters *n*_*i*_, *Cp*_*i*_ and *C*(1− *p*_*i*_) is fitted to the data. Here p_*i*_ is the mean variant frequency at locus *i* while *C*, learnt globally across loci, describes the extent of variance in allele frequency additional to that caused by the finite depth of sequencing *n*_*i*_. Second, the effective depth nei for each locus, derived from the binomial and beta-binomial standard deviation formulae (see Methods below), is calculated as

$$n_{i}^{e}=\frac{n_{i}(1+C)}{n_{i}+C}$$

This statistic describes the extent to which sequencing of a single sample represents the inferred mean frequency of each allele in a population, giving a conservative estimate of the variance arising from the sampling and sequencing process combined.

### 2.1 Application to simulated data: effect of limited viral load

As a preliminary step, we applied our statistic to simulated data describing the sequencing of a sample containing a limited number of viral particles. In this circumstance, sequencing of a small number of viral particles with a high read depth leads to the repeated sequencing of individual stretches of viral genetic material, limiting the extent of information available.

**Figure 2.**
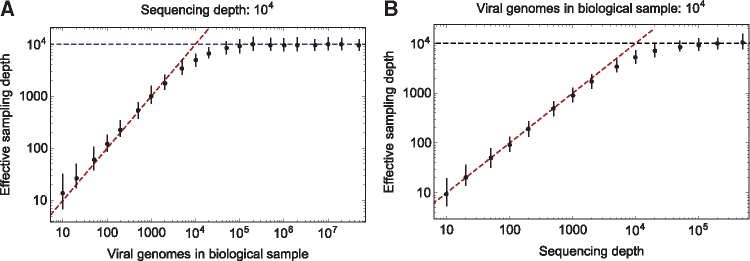
(A) Effective read depths given a sample of a finite number of viral particles and a constant read depth in sequencing of 10^4^ at each site. Black dots show harmonic mean effective read depths across a set of 100 sets of simulations, while error bars show 95% high and low ranges for this statistic. At low particle numbers, the inferred values are close to the red dashed line, which indicates equality between the effective read depth and the number of viral particles in the sample. At high particle numbers, the inferred values are close to the blue dashed line, which indicates equality between the effective, and absolute, read depths. More than 2 × 10^5^ particles were required to get a mean effective depth within 95% of the actual read depth. (B) Effective read depths given a sample of a fixed number of viral particles and a range of depths of sequencing.

To illustrate the effect of a limited sample, the effective read depth was calculated for a range of simulated populations with a constant read depth of 10^4^ and a varying number of viral genomes in the sample (Fig. 2A), and for a sample containing 10^4^ viral genomes with a varying depth of sequencing (Fig. 2B). Finite-depth sequencing of a limited number of viruses is essentially as a process of two successive sampling events, with depths equal to the number of particles in the sample and the number of reads collected in the sequencing of the variant site. As such, where the number of particles is considerably smaller than the sequencing depth, it is the number of particles that controls the effective sample depth. Conversely, where the number of particles is large, the effective sample depth approaches the number of reads at a locus. The rate at which the effective depth approaches this limit is slow; given a read depth of 10^4^, more than 2 × 10^5^ particles were required to obtain an effective depth within 95% of the depth-dependent limit.

### 2.2 Application to repeat-sequencing data

Application to repeat-sequencing data from viral populations gave a broad range of results. Four sets of data, comprising the repeat sequencing of single samples, were considered. In each set, data generated from distinct variations of the default protocol were compared, with replicate populations being split at different points in the process. Full details of each sample are given in Supplementary Tables S1–S4.

- HIV: Repeat sequencing of thirteen HIV samples. Nucleic acid extracts were split into replicates, then processed either using the default cDNA synthesis/SureSelect method (Brown et al. 2016) (see SureSelect target enrichment in Section 4), or using an alternative SureSelect RNA sequencing (see SureSelect RNA target enrichment in Section 4).
- Noro: Repeat sequencing of a single norovirus sample. During library preparation, the sample was split into two after enrichment, and the second PCR step was performed independently for each replicate. These second rounds of PCR involved either 18 or 22 rounds of amplification.
- HCV01: Repeat sequencing of four HCV samples. Nucleic acid extracts were split into replicate pairs, some of which were diluted and subjected to DNase digestion for depletion of human genomic material prior to cDNA synthesis.
- HCV02: Repeat sequencing of eleven HCV samples; repeat MiSeq runs were performed with replicate final libraries, with different loading concentrations of the final pooled library used in the sequencing process; this resulted in good and underclustered sequencing runs.

**Figure 3.**
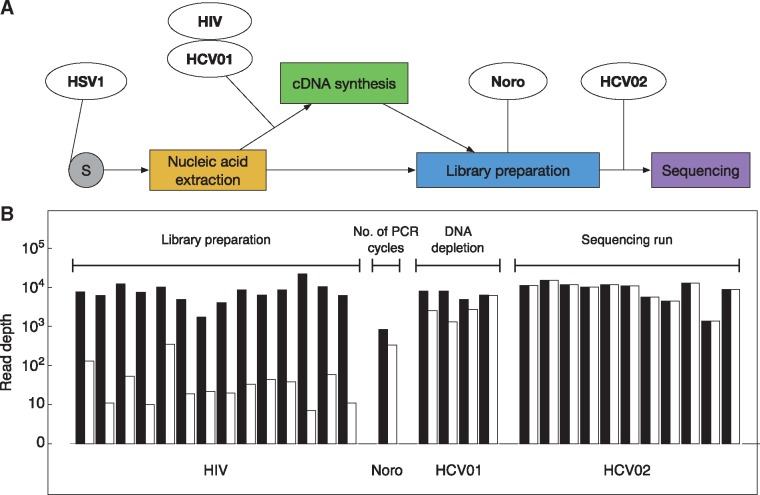
(A) Outline sequencing approach. In the standard protocol, cDNA was synthesised from RNA extracts prior to library preparation. Points at which the splitting of replicates occurred for different datasets in the study are marked. In the HSV1 set, independent replicate samples were collected. The HIV extracts were split with one aliquot processed by the standard cDNA synthesis/SureSelect method and the other processed with a SureSelect RNA sequencing approach. Depletion of host genomic DNA prior to cDNA synthesis was performed on aliquots of the HCV01 extracts. In the Noro set, replicates were split before the second round of PCR amplification during the library preparation. In the HCV02 set, the final library was split, then sequenced on two independent MiSeq runs. (B) Mean absolute (black) and effective (white) read depths for replicates within four sets of samples that have been repeatedly sequenced according to different protocols. Labels below each set of depths indicate the dataset; labels above each set indicate the mode of difference between replicates. In our approach, the two methods of processing replicate HIV samples produced inconsistent results, leading to low effective depths.

In our default process for RNA viruses, cDNA was synthesised from RNA extracts and library preparation performed with targeted enrichment using the SureSelect XT kit, which uses overlapping capture RNA baits complementary to, and spanning the length of, a specific pathogen genome. Sequencing was conducted using the Illumina MiSeq platform. Figure 3A shows an outline of the sequencing process, along with points of variation between datasets.

**Figure 4.**
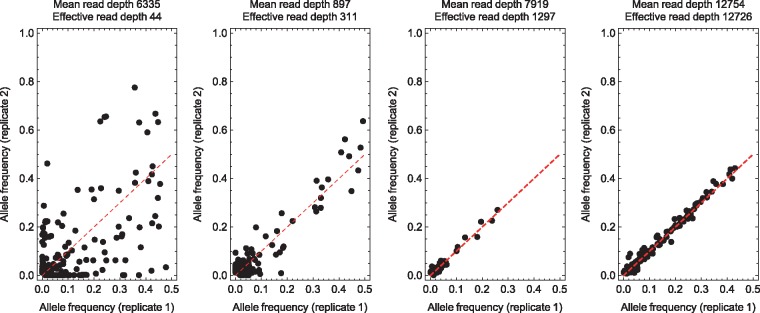
Allele frequencies derived for representative sets of viral samples. Data are shown for samples from the HIV, Noro, HCV01, and HCV02 sets, respectively. The frequency shown is that of the minority allele in the first replicate. The red dashed line shows perfect agreement between frequencies.

Calculations of effective read depths showed a range of results (Fig. 3B). Where, following library preparation, a sample was split and sequenced twice, allele frequencies were reproduced to a level of accuracy very close to that implied by the absolute read depth; in the HCV02 set effective read depths were uniformly within 99% of their corresponding absolute values. The difference in the results obtained via sequencing using a good or under-clustered MiSeq run was small; this verifies the ability of Illumina sequencing, given the same input material, to reproducibly capture genetic diversity. In other replicates, where some difference existed between experimental protocols, increased differences were seen. Dilution and DNA depletion of samples prior to library preparation led to a slight decrease in the effective, compared to the absolute read depth, with effective depths sometimes falling to a sixth of the absolute read depth. Replication of a PCR amplification step during library preparation also had a relatively small effect, reducing the effective read depth to a third of the absolute value. More dramatic differences in statistics were inferred for the HIV sequence data, for which the effective depths of sequencing were up to four orders of magnitude smaller than the absolute values. This result indicates that the different methods of processing the HIV samples gave inconsistent results; as we discuss below, the SureSelect RNA sequencing method used in this case did not give a good replication of the standard protocol. Illustrative figures showing allele frequencies for four representative datasets are shown in Fig. 4.

### 2.3 Application to replicate samples

Application of our approach to replicate samples also gave mixed results. Independent samples collected from a host at a single time-point give an independent replication of the data collection process, thereby providing a full estimate of the extent to which the within-host viral population is accurately described. We here considered two such datasets:

- HSV1: Repeat sequencing of two HSV1 datasets, containing two and three replicate samples of cerebrospinal fluid collected from the same host at the same time point.
- Flu: A/H1N1 influenza samples collected from different locations within ferrets a single day after infection (Lakdawala et al. 2015). Samples were collected from i) six locations in each of three ferrets: nasal wash, nasal turbinate, bronchoalveolar lavage, right and left lung, and soft palate; and ii) two locations in each of three ferrets: nasal turbinate and trachea.

**Figure 5.**
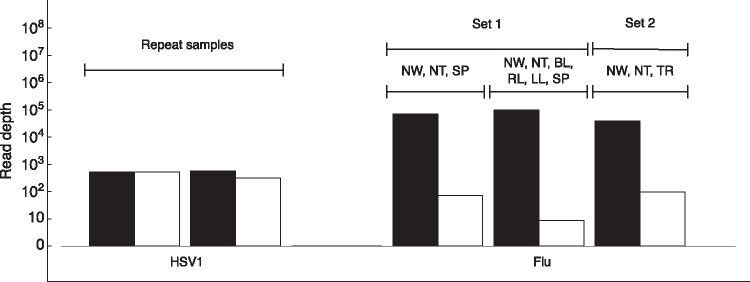
Mean absolute (black) and effective (white) read depths for replicates within four sets of samples that have been repeatedly sequenced according to different protocols. Labels below each set of depths indicate the dataset; labels above each set indicate the mode of difference between replicates. Codes refer to viruses collected via nasal wash (NW), nasal turbinate (NT), bronchaeolar lavage (BL), and from the soft palate (SP), right lung (RL), left lung (LL) and trachea (TR) of an animal.

Further information related to the HSV1 dataset is provided in Supplementary Table S5. Mean absolute and effective read depths for these populations are shown in Fig. 5. Data from the HSV1 sequencing gave high effective population sizes, suggesting a high degree of reproducibility between samples. By contrast the influenza data gave more complex results. Here, where data were collected from slightly different parts of each animal, differences between the samples reflect both error in the sequencing process (which may be negligible) and genuine differences between spatially distinct populations within the animal. The results here suggest that samples collected from the upper respiratory tract (nose, soft palate, trachea), exhibit greater consistency than samples collected from across upper and lower respiratory regions within the host.

The results from replicate sample data likely represent the extent of uniformity or non-uniformity in the genetic composition of the virus within-host. In the patients studied, the HSV1 population appears to be well-mixed in the cerebrospinal fluid at the time of sampling. By contrast, the low effective depths calculated for the influenza populations reflect differences in the population in different parts of the host; the population at one location is not representative of the population in another region within-host.

## 3. Discussion

We have here investigated the extent of similarity between allele frequencies derived from cases in which replication of all or part of the sequencing process was carried out on material from the same biological sample, and from cases in which sequencing was carried out on replicate biological samples from an individual. In each case, we identified a range of results. While each of our samples had an acceptably high absolute read depth, a high read depth did not guarantee a high extent of reproducibility between samples. Further, while the numbers of viral genomes in the samples sequenced in this study are broad estimates, rather than precise values, the estimates obtained did not correlate with data quality in an obvious way; high template number is not of itself a guarantee of high precision.

Different factors might be suggested to explain the results obtained. In the HCV02 samples, material was split post library preparation, with replicates sequenced in different MiSeq runs, albeit runs with different setup parameters. The high effective depths in this case likely reflect a high generic precision of genome sequencing technology. We note that, according to its definition, the effective read depth cannot exceed the absolute read depth; the values calculated here were close to that limit. In the case of the norovirus and HCV01 datasets, the difference in outcomes was slightly greater; the slightly different experimental procedures undergone by each of the replica sets induced small changes in the outcome. Finally, in the HIV dataset, where different procedures for library preparation were used, a greater difference in derived statistics was observed. Given previous validation of the default SureSelect approach (Thomson et al. 2016), it is likely that the SureSelect RNA sequencing approach produced an error in this instance. In other HIV sequencing studies, within-sample measures of allele frequency precision have indicated more precise characterisation of minor variant frequencies (Zanini et al. 2015); it should not be concluded from our result that viral sequencing from HIV samples is more problematic than from samples of any other virus. However, we note the general result that where an experimental method distorts the composition of a viral population, measures of sequencing precision generated from within a single sample could be misleading; internal consistency does not imply an accurate representation of the true viral population.

We have here been careful to distinguish between variance in a population arising from sequencing noise and that arising from genuine differences between biological samples. In the analysis of data from replicate samples, we show in the case of HSV-1 that repeat sampling from a host does not necessarily lead to inconsistent results. However, in the influenza population, where genuine differences in viral diversity exist in different parts of a host, a single sample did not give a good representation of the population as a whole. This provides another example in which within-sample measures of sequencing precision might not capture the extent of variation between the compositions of the sequenced material and the within-host viral population. The extent to which this factor affects sequence data will depend upon the biology of the virus being sampled, specifically upon the extent to which viruses within the host form a well-mixed population.

Accurate measurement of the consistency of data is of value in the quantitative analysis of genome sequence data, providing an estimate of the precision with which a dataset describing a viral population represents statistics describing that population. Where gross changes in allele frequency over time are being considered, an effective depth of 20 would be sufficient to distinguish high, low and intermediate frequencies. What might be termed a ‘bad’ dataset might reasonably be included in an analysis, so long as the uncertainty in the data is properly accounted for. In some cases, however, a high degree of precision about a population would be desirable. Where small changes in frequency can affect the result obtained, such as in the inference of transmission bottlenecks between hosts (Poon et al. 2016), verification of sequencing accuracy becomes of greater importance; a failure to account for noise in this case could cause an underestimate of the correct bottleneck size.

The key finding of our study is that care needs to be taken in evaluating genome sequence data from viral populations, particularly where genomic data is used to fit models of within-host viral evolution. While this conclusion is in many ways acknowledged in the previous literature, we note that methods for accounting for noise in sequence data, when used at all, do not often consider both sampling bias and the uncertainty in genome sequencing. In an ideal case, we propose that independent samples, collected contemporaneously from a host or evolutionary experiment, give the best measure of the precision with which population genetic statistics can be measured. Where studies set out to evaluate changes in viral populations, the collection and processing of replicate samples, sufficient to describe the extent of variation between them, would add a further degree of statistical verification. In cases where exact replica samples are not available, comparison between similar samples may provide an approximate measure. For example, where samples from a population are collected at closely separated points in time, the population will be altered between sampling times by evolutionary factors such as mutation, selection, and genetic drift. These factors are likely to increase the distance between the samples, leading to an artificially reduced effective read depth, and a conservative estimation of noise. However, in the absence of ideal data, proxy estimates of this form can provide a useful basis for evolutionary calculations (Illingworth 2015).

## 4. Methods

### 4.1 Preparation and sequencing of clinical samples

In our default protocol, cDNA was synthesised using the Sure Select approach. This approach eliminates PCR from the initial sample preparation and viral isolation step, and only includes PCR amplification during library preparation (30–34 cycles divided between two separate rounds of PCR amplification). Previous studies have shown this method to give a high degree of accuracy in reproducing viral diversity (Brown et al. 2016; Thomson et al. 2016). Approximate estimates of the number of pathogen genomes input into library preparation were calculated either from diagnostic values or Ct values.

#### 4.1.1 cDNA synthesis

RNA extracts were concentrated to 11 μl prior to first-strand cDNA synthesis. First-strand cDNA was synthesised using random primers and SuperScript III (SS III, Life Technologies) as per manufacturer‘s instructions. Briefly, 1 μl of 10 mM (each) dNTP mix and 1 μl of 3 μg/ml random primers were incubated with 11 μl RNA for five minutes at 65 °C to anneal primers to RNA template. RNA-primer templates were mixed with 4 μl 5× first-strand buffer, 1 μl 0.1 M DTT, 1 μl RNase OUT and 1 μl SS III at 25 °C for 5 minutes followed by cDNA synthesis at 50 °C for 1 hour and enzyme inactivation at 70 °C for 15 minutes. Second-strand cDNA was synthesised using second-strand cDNA Synthesis kit (NEB) as per manufacturer‘s instruction. Briefly, 20 μl first-strand cDNA was incubated with 48 μl water, 8 μl 10× second-strand buffer and 4 μl second-strand enzyme mix at 16 °C for 2.5 hours. Double-stranded cDNA was purified and concentrated with Genomic DNA Clean and Concentrator (Zymo Research), as per manufacturer‘s instructions, with a 30 μl elution volume and quantified with Qubit dsDNA high-sensitivity (HS) kit (Invitrogen).

#### 4.1.2 DNase depletion method

HCV replicate samples in the dataset HCV01 were sequenced as part of optimizations to improve the efficiency of cDNA synthesis by treating clinical RNA extracts with DNase to remove residual host-derived gDNA. DNase treatment and cleanup was performed using the RNA Clean & Concentrator kit (Zymo Research, CA, USA). Briefly, diluted RNA extracts for two samples were split into equal 50 μl aliquots, each of which was combined with 100 μl RNA binding buffer, followed by addition of 150 μl 95% ethanol and centrifugation through a spin column, and washing. DNase treatment was performed on the column according to the manufacturer‘s instructions for one aliquot, with the control aliquot being treated in parallel with DNase buffer without enzyme. After digestion, samples were subjected to three rounds of washing according to the manufacturer‘s instructions, before elution into 15 μl nuclease-free water. Following elution, samples were diluted to 50 μl and we proceeded to cDNA synthesis as described above.

### 4.2 SureSelect target enrichment: RNA baits design

Overlapping 120-mer RNA baits complimentary to and spanning the length of specific pathogen genomes were designed using an in-house PERL script. The specificity of the baits was verified by BLASTn searches against the Human Genomic + Transcript database. The custom-designed bait libraries were uploaded to E-array and synthesised by Agilent Biotechnologies.

### 4.3 SureSelect target enrichment: library preparation, hybridisation and enrichment

DNA samples were quantified and carrier G147 Human Genomic DNA: male (Promega) was added if necessary to obtain a total of 200 ng. All DNA samples were sheared for 150 seconds using a Covaris E210 (duty cycle 5%, PIP 175 and 200 cycles per burst). End-repair, non-templated addition of 3’-A adapter ligation, hybridisation, PCR and all post-reaction clean-up steps were performed according to the SureSelect Illumina Paired-End Sequencing Library XT protocol. All recommended quality steps were performed.

### 4.4 SureSelect RNA target enrichment: library preparation, hybridisation and enrichment

In the SureSelect RNA sequencing runs, all RNA samples were dried by vacuum centrifugation before resuspension using 19 μl RNASeq Fragmentation Mix. Fragmentation, generation of adapter-ligated cDNA libraries, hybridisation, PCR and all post-reaction clean-up steps were performed according to the SureSelectXT Automated RNA Target Enrichment protocol. All recommended quality steps were performed.

### 4.5 Estimated numbers of viral genomes

Rough estimates for the numbers of genomes in each sample were calculated for the samples collected in this study. Given diagnostic copy number estimates, these values were converted into copy numbers in the eluted volume after extraction. For the manual cDNA synthesis, it was assumed that 100% of this was converted by the synthesis process into DNA (i.e. DNA copies). The number of copies going into library prep were then based upon the volume of cDNA added. For the SureSelect RNA sequencing protocol the number of copies going into the library prep were calculated based upon the volume of cDNA added. For the HSV1 data, Ct values from qPCR are provided. We acknowledge that the resulting values are highly approximate; these are provided as guideline figures only.

### 4.6 Processing sequence data

For each sequence dataset, bioinformatic tools were used to identify a reference sequence (Yamashita et al. 2016) align short reads (Li and Durbin 2009), filter the data (bases selected for PHRED score ≥ 30) and calculate allele frequencies at polymorphic loci (Brown et al. 2016). Effective sequencing depth calculations were conducted over variants reaching a frequency of 1% in at least one sample, and with a minimum read depth of 100 in each sample considered.

### 4.7 Derivation of the effective depth of sequencing

We consider sets of replicate allele frequencies, derived from a population. Assuming that two nucleotides are observed at a given locus, we denote the frequency of the minority allele at locus *i* in replicate *r* by *p*^*r*^*_i_*, while the total number of observations at this locus are given by the read depth *n^r^_i_*. For each variant, an estimate of the true allele frequency is calculated across all replicates as

$$p_{i}=\frac{\sum_{r}n_{i}^{r}p_{i}^{r}}{\sum_{r}n_{i}^{r}}$$

A set of distributions are then fitted to the data across each replicate. A standard beta-binomial distribution is parameterised as having the probability density function

$$f(k|n,\alpha ,\beta )=\left(\begin{array}{c} n\\ k \end{array}\right)\frac{B(k+\alpha ,n-k+\beta )}{B(\alpha ,\beta )}$$

where *B* represents the beta function. Here, data for the locus *i* is represented by such a model, setting parameters α = *Cp*_*i*_ and β = *C*(1 − *p*_i_). Under this formulation, the mean of the distribution is the simple product n*p_i_*, while *C* determines the variance of the model; the log likelihood of the model given the data at this locus is

$$L_{i}=\sum_{r}\left(\begin{array}{c} n_{i}^{r}\\ k_{i}^{r} \end{array}\right)\frac{B(k_{i}^{r}+Cp_{i},n_{i}^{r}-k_{i}^{r}+C(1-p_{i}))}{B(Cp_{i},C(1-p_{i})}$$

where *k_i_^r^* = *n^r^_i_ p^r^_i_.* Within this model, all parameters are defined by the data except for the parameter *C*, for which a maximum likelihood value is obtained across the data for all loci. Given a maximum likelihood value for the parameter *C*, we note that the SD for the allele frequency at locus *i* in replica *r* is given by

$$\sqrt{\frac{p_{i}(1-p_{i})(C+n_{i}^{r})}{n_{i}^{r}(C+1)}}$$

*n^r^_i_*(C + 1) We derive the effective read depth for this variant in this replicate as the value *n* that would give the same variance were the sample drawn from a standard binomial equation, that is,

$$\sqrt{\frac{p_{i}(1-p_{i})}{n_{i}^{e}}}=\sqrt{\frac{p_{i}(1-p_{i})(C+n_{i}^{r})}{n_{i}^{r}(C+1)}}$$

giving

$$n_{i}^{e}=\frac{n_{i}^{r}(1+C)}{n_{i}^{r}+C}$$

### 4.8 Simulation of variant allele frequencies

Simulated allele frequencies from populations with a range of viral loads were generated from the single-locus equilibrium distribution of the neutral Wright-Fisher process, which, given a population with effective size N_*e*_ and mutation rate μ, is specified by

$$P(x)\propto {\left(1-x\right)}^{2N_{e}\mu -1}x^{2N_{e}\mu -1}e^{2N_{e}x}$$

A total of 500 variant frequencies were generated from this distribution, using values N_*e*_ = 113.8, μ = 2 × 10^−5^, inferred for influenza populations (Bedford et al. 2010; Sanjuan et al. 2010) to get a reasonable distribution of frequencies. The underlying population from which viruses were collected was assumed to be large.

To simulate the effect of limited viral load on the precision of viral sequencing, a sample of *v* viruses were collected from the population, each virus having variant alleles with probabilities determined by the population allele frequencies. To simulate sequencing of read depth *d*, a total of *d* viruses were sampled from the population, with replacement, calculating observed allele frequencies from these reads. The frequencies of variant alleles which appeared in each of the two simulated replica populations at a frequency greater than zero were used to calculate an effective read depth.

In Fig. 1, where mean effective sample depths are calculated across multiple simulations, the harmonic mean of the inferred values is described. That is, for the set {*n_i_*}^*k*^, we report

$$\frac{1}{k}\sum_{i=1}^{k}n_{i}$$

This is consistent with calculations of effective population size across multiple generations. In the reports of effective sample depths calculated for real datasets, variation in the absolute sample depths have a strong influence in the values inferred genome wide. The arithmetic mean values of the absolute and effective sample depths, measured across loci, are reported. 

## Supplementary data

Supplementary data are available at *Virus Evolution* online.

**Conflict of interest:** None declared.

## Author contributions statement

C.J.R.I. conceived the idea for the project, wrote code, and performed calculations. C.J.R.I. and R. W. wrote the manuscript. Sequencing experiments and data curation were performed by S.R., M.A.B., H. T. and R.W, while J.B. oversaw the process of data collection and curation. All authors reviewed the manuscript.

## Additional information

Accession numbers: All sequence data generated in the course of this study is available from the SRA database (accession number PRJNA380188).

The SAMFIRE software package is available from https://github.com/cjri/SAMFIRE.

Simplified code for calculating effective sequence depths is available from https://github.com/cjri/CalcDepthEF.

## Supplementary Material

Supplementary Table 1

Supplementary Table 2

Supplementary Table 3

Supplementary Table 4

Supplementary Table 5
