# Supplementary material for: On the effective depth of viral sequence data
Source: Virus Evol. 2017 Nov 14;3(2):vex030. doi: 10.1093/ve/vex030 (PMC5724399; doi:10.1093/ve/vex030)
Supplement: Supplementary Table 1 [file vex030_supp_tables1.pdf]

Dataset	Replica set	ID	Replica	Original sample Type	Diagnostic Pathogen load IU/ml	Extraction method	Volume extracted uL	Elution Volume uL	Library Prep Protocol	Estimated target genomes input	No. of pre-hyb PCR cycles	No. of post-hyb PCR cycles	Mean read depth following alignment
HIV	1	HIV1_2176	1	Plasma	225987	Qiagen DSP Virus pathogen Midi kit	1000	110	cDNA synthesis followed by standard SureSelectXT 200 ng protocol	16424	12	18	8605
			2						RNA input into Agilent SureSelectXT RNA protocol	70389		22	6377
	2	HIV1_2184	1		322841				cDNA synthesis followed by standard SureSelectXT 200 ng protocol	20076		18	4496
			2						RNA input into Agilent SureSelectXT RNA protocol	100379		22	7111
	3	HIV1_2188	1		5300788				cDNA synthesis followed by standard SureSelectXT 200 ng protocol	329925		18	7506
			2						RNA input into Agilent SureSelectXT RNA protocol	1649623		18	16673
	4	HIV1_2196	1		5834				cDNA synthesis followed by standard SureSelectXT 200 ng protocol	1462		18	10059
			2						RNA input into Agilent SureSelectXT RNA protocol	1814		18	106
	5	HIV1_2199	1		4971483				cDNA synthesis followed by standard SureSelectXT 200 ng protocol	360344		18	9061
			2						RNA input into Agilent SureSelectXT RNA protocol	1544333		18	10622
	6	HIV1_2200	1		133396				cDNA synthesis followed by standard SureSelectXT 200 ng protocol	9678		18	2574
			2						RNA input into Agilent SureSelectXT RNA protocol	41476		22	6256
	7	HIV1_2204	1		38153				cDNA synthesis followed by standard SureSelectXT 200 ng protocol	2765		18	615
			2						RNA input into Agilent SureSelectXT RNA protocol	11852		22	1718
	8	HIV1_2207	1		39286				cDNA synthesis followed by standard SureSelectXT 200 ng protocol	2441		18	2953
			2						RNA input into Agilent SureSelectXT RNA protocol	12204		22	4599
	9	HIV1_2208	1		144655				cDNA synthesis followed by standard SureSelectXT 200 ng protocol	9011		18	7058
			2						RNA input into Agilent SureSelectXT RNA protocol	45056		22	9573
	10	HIV1_2218	1		82080				cDNA synthesis followed by standard SureSelectXT 200 ng protocol	7695		18	8394
			2						RNA input into Agilent SureSelectXT RNA protocol	25650		22	3473
	11	HIV1_2221	1		368546				cDNA synthesis followed by standard SureSelectXT 200 ng protocol	22959		18	10037
			2						RNA input into Agilent SureSelectXT RNA protocol	114793		22	6787
	12	HIV1_2223	1		3799				cDNA synthesis followed by standard SureSelectXT 200 ng protocol	592		18	36417
			2						RNA input into Agilent SureSelectXT RNA protocol	1183		18	407
	13	HIV1_2225	1		42622655				cDNA synthesis followed by standard SureSelectXT 200 ng protocol	3569501		18	5515
			2						RNA input into Agilent SureSelectXT RNA protocol	13385627		18	12256
