# Supplementary material for: On the effective depth of viral sequence data
Source: Virus Evol. 2017 Nov 14;3(2):vex030. doi: 10.1093/ve/vex030 (PMC5724399; doi:10.1093/ve/vex030)
Supplement: Supplementary Table 2 [file vex030_supp_tables2.pdf]

Dataset	Replica set	ID	Replica	Original sample Type	Diagnostic CT	Volume extracted uL	Elution Volume uL	Library Prep Protocol	Estimated target genomes input	No. of pre-hyb PCR cycles	No. of post-hyb PCR cycles	Mean read depth following alignment
Noro	1	NOR_2048	1<br>2	Faeces	25	200	90	cDNA synthesis followed by standard SureSelectXT 200 ng protocol	1800	12<br>12	18<br>22	296<br>1429
