# Supplementary material for: On the effective depth of viral sequence data
Source: Virus Evol. 2017 Nov 14;3(2):vex030. doi: 10.1093/ve/vex030 (PMC5724399; doi:10.1093/ve/vex030)
Supplement: Supplementary Table 3 [file vex030_supp_tables3.pdf]

Dataset	Replica set	ID	Replica	Original sample Type	Extraction method	Volume extracted uL	Elution Volume uL	Diagnostic Pathogen load IU/ml	Pre-library prep dilution	DNA depletion	Library Prep Protocol	No. of pre-hyb PCR cycles	No. of post-hyb PCR cycles	Mean read depth following alignment
HCV01	1	HCV_2378	1	Plasma	Qiasymphony DSP Midi Kit Virus pathogen Version 1	1000	100	6243148	None	Yes	cDNA synthesis followed by standard SureSelectXT 200 ng protocol	12	18	11675
		HCV_2379	2						1/100	Yes				7817
		HCV_2380	3						1/10K	Yes				71
		HCV_2386	4						None	No				10842
		HCV_2387	5						1/100	No				4398
		HCV_2388	6						1/10K	No				80
	2	HCV_2384	1					56051	None	Yes				8032
		HCV_2392	2						None	No				7549
	3	HCV_2381	1					2082499	None	Yes				5761
		HCV_2382	2						1/100	Yes				2647
		HCV_2389	3						None	No				7089
		HCV_2390	4						1/100	No				2147
	4	HCV_2385	1					367817	None	Yes				5769
		HCV_2393	2						None	No				6829
