# Supplementary material for: On the effective depth of viral sequence data
Source: Virus Evol. 2017 Nov 14;3(2):vex030. doi: 10.1093/ve/vex030 (PMC5724399; doi:10.1093/ve/vex030)
Supplement: Supplementary Table 4 [file vex030_supp_tables4.pdf]

Dataset	Replica set	ID	Replica	Original sample Type	Extraction method	Volume extracted uL	Elution Volume uL	Diagnostic Pathogen load IU/ml	Estimated target genomes input	Library Prep Protocol	No. of pre-hyb PCR cycles	No. of post-hyb PCR cycles	Cluster density (k/mm^2)	Mean read depth following alignment
HCV02	1	HCV_2001	1	Plasma	Qiasymphony DSP virus/pathogen mini kit Version 1	200	60	5.16E+06	1702800	cDNA synthesis followed by standard SureSelectXT 200 ng protocol	12	18	931	19650
			2										162	2492
	2	HCV_2003	1					5.24E+05	326627				931	26502
			2										162	3476
	3	HCV_2004	1					1.67E+06	979733				931	20357
			2										162	2559
	4	HCV_2005	1					4.87E+07	19642333				931	17823
			2										162	2297
	5	HCV_2007	1					1.00E+07	5500000				931	19844
			2										162	2547
	6	HCV_2008	1					8.13E+05	417340				931	19298
			2										162	2432
	7	HCV_2009	1					5.95E+05	261800				931	9779
			2										162	1322
	8	HCV_2011	1					4.27E+04	28182				931	7571
			2										162	1180
	9	HCV_2013	1					2.20E+06	1290667				931	21622
			2										162	2864
	10	HCV_2014	1					1.30E+05	61967				931	1758
			2										162	324
	11	HCV_2016	1					1.00E+06	586667				931	14602
			2										162	1957
