# Supplementary material for: On the effective depth of viral sequence data
Source: Virus Evol. 2017 Nov 14;3(2):vex030. doi: 10.1093/ve/vex030 (PMC5724399; doi:10.1093/ve/vex030)
Supplement: Supplementary Table 5 [file vex030_supp_tables5.pdf]

Dataset	Replica set	ID	Replica	Original sample Type	Extraction method	Volume extracted ul	Elution volume ul	Diagnostic Ct value	Library Prep Protocol	Mean read depth following alignment
HSV1	1	C4	1	Cerebrospinal fluid	QIAMP DNA Mini	200	100	30.19	standard SureSelectXT 200 ng protocol	857
			2			135		32.75		273
			3			100		30.39		299
	2	C5	1			400	100	27.47		673
			2			60		31.35		384
